# Prolonged Tricyclic Antidepressant Toxicity in a Pediatric Patient: The Importance of Understanding the Multifactorial Nature of Amitriptyline Metabolism

**DOI:** 10.7759/cureus.112541

**Published:** 2026-07-12

**Authors:** Ashleigh W Navarro, Hanna Sahhar

**Affiliations:** 1 Pediatrics, Edward Via College of Osteopathic Medicine, Spartanburg, USA; 2 Pediatric Intensive Care Unit, Spartanburg Regional Healthcare System, Spartanburg, SC, USA

**Keywords:** amitriptyline, cannabinoid pharmacology, cyp-mediated interactions, effects of bmi on medications, pediatric overdose, tricyclic antidepressant toxicity

## Abstract

Tricyclic antidepressant (TCA) overdose is a potentially life-threatening condition characterized by anticholinergic, cardiotoxic, and neurotoxic effects. Amitriptyline is a highly lipophilic TCA metabolized by cytochrome (CYP)2C19, CYP3A4, and CYP2D6 and may be associated with altered metabolism with concurrent use of cannabinoids, mutations of the CYP enzymes, and elevated body mass index (BMI). With higher levels of adipose tissue, elevated BMI can lead to a larger volume of distribution for lipophilic drugs. Deviations from expected drug clearance may prolong toxicity and complicate management.

We describe a 15-year-old female with obesity (BMI of 36 kg/m²) and a complex psychiatric history who presented after an intentional overdose of approximately 750 mg of amitriptyline. On arrival, she was unresponsive with a Glasgow Coma Scale (GCS) score of 3, tachycardia, respiratory distress, and electrocardiographic evidence of TCA cardiotoxicity, including a wide complex tachycardia and QRS prolongation. She required emergent intubation, sodium bicarbonate therapy, seizure prophylaxis, serial electrocardiograms, laboratory monitoring, and intensive care monitoring. Despite appropriate supportive management and based on typical half-life estimates under therapeutic conditions, QRS prolongation persisted, and serum amitriptyline/nortriptyline levels remained markedly elevated on day seven (combined level 612 ng/mL) and detectable on day ten (combined level 131 ng/mL).

The prolonged elimination observed in this case was likely multifactorial, with cannabinoid use potentially contributing to prolonged toxicity through interactions with CYP enzymes. Additional contributing factors may have included elevated BMI or CYP enzyme polymorphisms. Although CYP testing was not performed in this case, consideration of testing in similar presentations in the future may aid in clinical management if a mutation is identified. The contribution of elevated BMI in this case remains speculative, as current evidence regarding its effects on the pharmacokinetics of lipophilic drugs is inconsistent, highlighting the importance of further studies on this topic.

This case illustrates the impact of cannabinoid use and other factors on the pharmacokinetics of amitriptyline and underscores the need for prolonged monitoring, serial electrocardiograms, and consideration of extended drug clearance in cases of pediatric patients with TCA overdose. Clinicians should be aware that standard half-life estimates may underestimate the duration of toxicity in populations that have factors influencing metabolism.

## Introduction

Tricyclic antidepressants (TCAs) are prescribed for major depressive disorder, anxiety, neuropathic pain, and migraine prophylaxis. They exert their primary therapeutic effect by inhibiting the reuptake of serotonin, norepinephrine, and, to a lesser extent, dopamine at presynaptic terminals in the central nervous system, thereby increasing synaptic concentrations of these neurotransmitters [1,2]. TCAs also act as antagonists at postsynaptic α-adrenergic, muscarinic, and histamine (H₁) receptors, contributing to their characteristic side effect profile [1].

Amitriptyline is a widely used TCA that undergoes extensive hepatic biotransformation via the cytochrome P450 (CYP) system [3]. Cytochrome (CYP)2C19 and CYP2D6 primarily metabolize it [3]. CYP2C19 converts amitriptyline to nortriptyline, an active metabolite with antidepressant properties, and nortriptyline is routinely measured along with amitriptyline when serum TCA levels are obtained. CYP2D6 further metabolizes amitriptyline and nortriptyline to less active metabolites such as 10-hydroxyamitriptyline, which subsequently undergo glucuronidation in the liver [4]. The elimination half-life of amitriptyline is reported to be 10-28 hours, while nortriptyline has a half-life of 16-80 hours [2].

A variety of factors can affect the pharmacokinetics of amitriptyline, including age, hepatic function, concomitant medications that induce or inhibit CYP enzymes, and genetic polymorphisms [5]. Amitriptyline is slowly absorbed from the gastrointestinal tract and typically reaches peak plasma concentrations within four to eight hours after ingestion [2]. It is highly lipophilic and widely distributed in tissues, with significant binding to plasma and tissue proteins [2].

Clinically, amitriptyline toxicity manifests along several pathways. Anticholinergic effects include blurred vision, dry mouth, urinary retention, sedation, and hypotension [4,6]. Cardiovascular toxicity becomes particularly concerning at plasma concentrations greater than 300 ng/mL and includes tachyarrhythmias, QRS prolongation, and risk of ventricular tachycardia (VT), ventricular fibrillation (VF), or torsades de pointes [6,7]. Electrocardiogram (ECG) findings classically show sinus tachycardia, QRS duration >100 ms, and possible arrhythmias [7]. Neurologic manifestations include decreased level of consciousness, respiratory depression, agitation, and seizures [7,8].

We present a case of severe, prolonged amitriptyline toxicity in an adolescent, highlighting altered pharmacokinetics in the context of cannabinoid use and possibly other factors altering metabolism, which emphasizes the importance of recognizing potential drug interactions that may contribute to prolonged toxicity.

## Case presentation

A 15-year-old female with a significant psychiatric history presented to the emergency department (ED) via emergency medical services after an intentional overdose. According to her mother, she ingested approximately 30 tablets of amitriptyline 25 mg (total estimated dose 750 mg) in a suicide attempt. Patient weighed 109 kilograms (kg) which equates to a BMI of 36 kg/m2 (calculated based on recorded height and weight).

Her past medical history included generalized anxiety disorder, major depressive disorder, migraines, previously diagnosed borderline personality disorder, and prior suicide attempts. Home medications included amphetamine/dextroamphetamine salts, sumatriptan, and venlafaxine. Immunizations were up to date.

Emergency department presentation

On arrival to the ED, the patient was unresponsive. Ingestion was one hour and 20 minutes before arrival to the ED. One dose of naloxone 1 mg was empirically administered intravenously without improvement in mental status. Initial vital signs were as follows: blood pressure 137/85 mm Hg, heart rate 132 beats per minute, oxygen saturation 96% on supplemental oxygen through a non-rebreather mask, and weight 109 kg, corresponding to a BMI of 36 kg/m².

Physical examination revealed tachycardia and signs of respiratory distress. Multiple healed superficial lacerations were noted on both upper extremities and the lateral neck. Neurologically, she had a Glasgow Coma Scale (GCS) of 3 with absent gag reflex, prompting preparation for emergent intubation. Pupils were 3 mm and sluggishly reactive.

**Figure 1:**
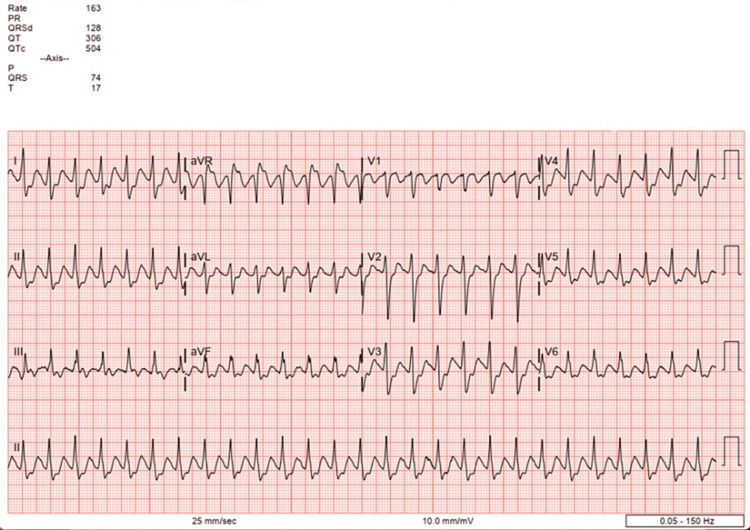
ECG on Day 0 (Presentation) Electrocardiogram obtained at ED presentation demonstrating a wide-complex tachycardia with ventricular rate of 163 beats per minute, prolonged QRS duration of 128 ms, and QTc of 504 ms, consistent with tricyclic antidepressant cardiotoxicity.

Initial ECG demonstrated a wide-complex tachycardia with a ventricular rate of 163 beats per minute and normal axis (Figure 1). The QRS duration was prolonged at 128 ms (normal: 80-100 ms), and the corrected QT interval (QTc) was 504 ms. Given these findings, two ampules of sodium bicarbonate at 50 mEq each and one dose of magnesium sulfate 2 g were administered emergently before intubation.

Poison Control was contacted and recommended aggressive supportive management, monitoring for serotonin syndrome, and initiation of a sodium bicarbonate infusion if QRS prolongation persists or if pH is <7.45, with the therapeutic goal being mild alkalinization

Initial laboratory evaluation

Initial laboratory workup was obtained in the ED at the time of initial presentation and intravenous (IV) placement, with the results summarized in Table 1. Arterial blood gas (ABG) was obtained after intubation and revealed a mild respiratory acidosis [pH 7.341, partial pressure of carbon dioxide (pCO₂) 46.5 mm Hg]. Complete blood count was unremarkable. Comprehensive metabolic panel findings included a low blood urea nitrogen (BUN) level, a low BUN/creatinine ratio, and mild hyperglycemia. Liver function tests were within normal limits. Urine drug screen was positive for barbiturates and cannabinoids; acetaminophen, salicylate, and ethanol levels were unremarkable. Urinalysis was normal and did not show any signs of co-ingestion with amphetamines since amphetamines were negative. No test or screen is available for Venlafaxine, but no reason to suggest co-ingestion, as no missing tablets were found from the patient’s prescribed meds. Given the presence of respiratory acidosis and QRS prolongation, a continuous sodium bicarbonate infusion was initiated.

**Table 1:** Emergency Department Laboratory Values ABG: arterial blood gas; BUN: blood urea nitrogen; ECG: electrocardiogram, pCO_2_: partial pressure of carbon dioxide, pO_2_: partial pressure of oxygen, HCO_3_-: sodium bicarbonate.

Test	Patient Result	Reference Range
Complete Blood Count		
White blood cells (cells/µL)	7.0 × 10³	4.5–10.0 × 10³
Red blood cells (millions/µL)	5.12	4.2-5.4
Hemoglobin (g/dL)	14.4	12.1-15.1
Hematocrit (%)	43.8	36.1-44.3
Mean corpuscular volume (fL)	85.6	80-100
Platelets (×10³/µL)	172	150-450
Comprehensive Metabolic Panel		
Sodium (mEq/L)	138	135-145
Potassium (mEq/L)	3.6	3.7-5.2
Chloride (mEq/L)	105	96-106
Bicarbonate (mEq/L)	24	23-29
Anion gap (mEq/L)	9	4-12
Osmolality (calculated, mOsm/kg)	273.66	275-295
BUN (mg/dL)	4	6-20
Creatinine (mg/dL)	0.74	0.6-1.3
BUN / Creatinine ratio	5.41	10-24
Glucose (mg/dL)	118	70-100
Calcium (mg/dL)	11.2	8.5-10.2
Corrected calcium (mg/dL)	11	8.5-10.2
Total protein (g/dL)	7.3	6.0-8.3
Albumin (g/dL)	4.3	3.4-5.4
Alanine aminotransferase (U/L)	11	4-36
Aspartate aminotransferase (U/L)	15	8-33
Alkaline phosphatase (U/L)	90	20-130
Total bilirubin (mg/dL)	0.5	0.1-1.2
Arterial Blood Gas		
pH	7.341	7.35-7.45
pCO₂ (mmHg)	46.5	35-45
pO₂ (mmHg)	86	75-100
HCO₃⁻ (mEq/L)	25.1	22-26
Oxygen saturation (%)	96	94-100
Toxicology and Others		
Acetaminophen	<10.0	0
Ethanol	Undetectable	<10
Salicylate Level	<2.5	<2.5
Creatine kinase (U/L)	52	30-135
Triglycerides (mg/dL)	109	<90
Magnesium (mg/dL)	2.1	1.7-2.2
Urine drug screen		
Barbituates	Positive	Negative
Cannabinoids	Positive	Negative

Pediatric intensive care unit course

The patient was admitted to the pediatric intensive care unit (PICU) on hospital day one. She remained intubated, deeply sedated with a propofol infusion, and had no spontaneous respiratory effort. Pupils were 3 mm and sluggishly reactive. She continued to be tachycardic. Intravenous fluids (5% dextrose with 40 mEq potassium chloride and 150 mEq sodium bicarbonate) were administered at 125 mL/hour. Midazolam was used adjunctively for sedation and seizure prophylaxis.

Management

The patient was managed with continuous sodium bicarbonate infusion to maintain the serum pH in the upper normal range and facilitate narrowing of the QRS interval. Serial electrocardiograms (ECGs) were obtained every six hours, and arterial blood gas analyses with serum electrolyte measurements were performed at six-hour intervals to guide ongoing management. A Foley catheter was placed to allow for strict intake and output monitoring. Overnight, the patient experienced one generalized myoclonic seizure, which resolved without additional complications.

On hospital day two, she remained intubated and tachycardic with urine output <0.5ml/kg for the first six hours and no stool output. She demonstrated improved responsiveness to suctioning, and pupils were 4 mm and reactive. Serial ECGs showed persistent QRS prolongation ranging from 108-115 ms. Sodium bicarbonate infusion, sedation, and supportive care were continued.

On hospital day three, vital signs remained stable. Neurologic status improved further, with responsiveness to deep suctioning and spontaneous respiratory effort. Pupils were 6 mm and reactive bilaterally. Considering improved mental status and respiratory drive, she was successfully extubated. ECGs continued to demonstrate QRS prolongation, with the most severe QRS duration of 119 ms. Sodium bicarbonate therapy was maintained.

**Figure 2:**
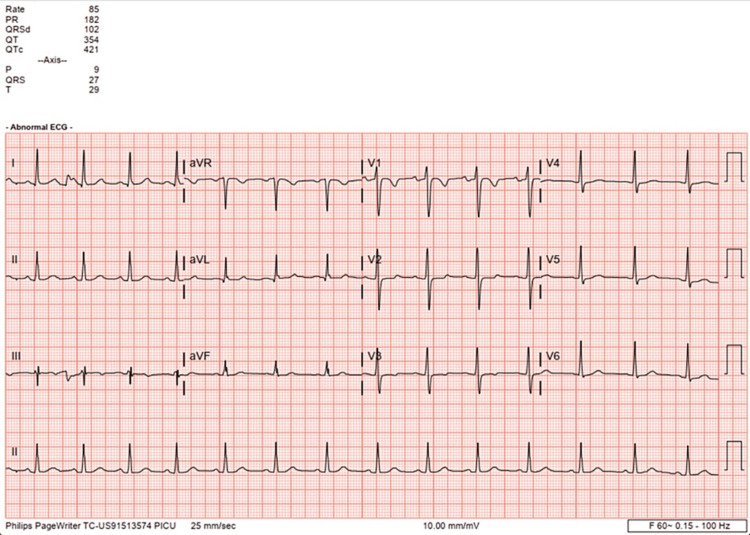
ECG on Day 6 of Hospitalization Electrocardiogram on hospital day 6 showing persistent QRS prolongation with QRS duration of 102 ms, prompting measurement of serum amitriptyline and nortriptyline levels.

During hospital days four and five, the patient remained hemodynamically stable. Sodium bicarbonate infusion was titrated based on ECG findings, with continuation when QRS duration exceeded 100 ms. Magnesium sulfate was administered if QTc exceeded 480 ms. She underwent electroencephalography and brain imaging, which did not reveal acute structural abnormalities. Serial ECGs continued to show QRS prolongation. She was transferred from the PICU to the general pediatric floor on hospital day five. On hospital day six, an ECG showed a QRS duration of 102 ms (Figure 2). Due to persistent ECG evidence of TCA effect, serum TCA levels were obtained.

Serum tricyclic antidepressant levels and subsequent course

On hospital day seven, the combined amitriptyline/nortriptyline level was 612 ng/mL (amitriptyline 301 ng/mL, nortriptyline 311 ng/mL; reference combined therapeutic range 80-200 ng/mL). QRS prolongation persisted on ECGs through hospital days seven and eight.

**Figure 3:**
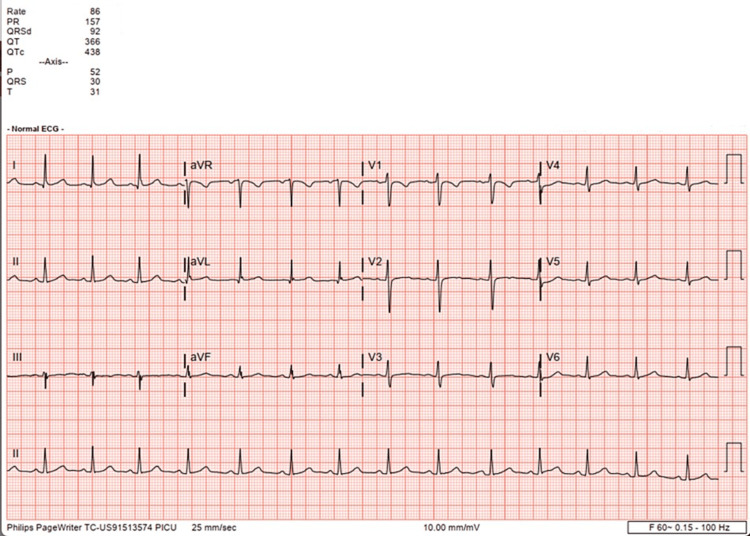
ECG on Day 9 of Hospitalization Electrocardiogram on hospital day 9 demonstrating normal sinus rhythm at 99 beats per minute and normalization of QRS duration to 91–92 ms following prolonged sodium bicarbonate therapy and gradual drug clearance.

On hospital day nine, ECG demonstrated normal sinus rhythm with a ventricular rate of 99 beats per minute and a normalized QRS duration of 91-92 ms (Figure 3). With normalization of the QRS interval, sodium bicarbonate therapy was discontinued.

On hospital day 10, before discharge, repeat TCA levels showed persistent but near-therapeutic combined levels (amitriptyline 59 ng/mL, nortriptyline 72 ng/mL; combined 131 ng/mL). The patient remained hemodynamically stable without recurrent neurologic or cardiac events and was deemed medically cleared for transfer to inpatient psychiatric care.

The patient's presentation can be described within the International Classification of Functioning, Disability, and Health (ICF) framework, demonstrating acute impairment in body functions secondary to amitriptyline toxicity.

## Discussion

This case illustrates significant deviation from expected amitriptyline pharmacokinetics and pharmacodynamics in an adolescent following intentional overdose. The patient exhibited classic features of TCA toxicity, including altered mental status, seizures, sinus tachycardia, and QRS prolongation [9].

TCAs block fast sodium channels in myocardial cells, slowing phase 0 depolarization and delaying conduction through the His-Purkinje system and ventricular myocardium [9]. This manifests on ECG as QRS prolongation and predisposes to potentially lethal ventricular arrhythmias, including VT and VF. QRS duration is widely accepted as a clinical marker of severity in TCA toxicity, with QRS duration [AW1] >100 ms associated with increased risk of seizures and ventricular arrhythmias [9]. TCAs are associated with an increased risk of seizure in patients with prolonged QRS complexes. It has been shown in the literature that plasma concentrations greater than 1000µg/l could also be an indicator of an increased risk, but QRS prolongation is a better predictor of the risk of seizures and cardiac arrest [8].

In this case, the patient’s prolonged course of cardiotoxicity and elevated TCA serum levels were notable despite the passage of sufficient time for expected drug clearance based on standard half-life estimates. Under usual conditions, a drug is considered essentially cleared after approximately five half-lives. Assuming a half-life of 10-28 hours for amitriptyline in a normal metabolizer [2], the ingested dose would be expected to be substantially eliminated by day five post-ingestion. However, ECG abnormalities persisted beyond this period, and toxic serum TCA levels were still present on day seven. It is important to note that the prolonged toxicity may reflect known overdose kinetics, but it is also important to consider other factors that may also influence metabolism.

Impact of cannabinoids on amitriptyline pharmacokinetics

Amitriptyline is broken down to the active metabolite nortriptyline through the CYP2C19 enzyme, and then further metabolism is done via hydroxylation of both amitriptyline and nortriptyline via CYP2D6 [3]. 

Studies have shown that use of cannabinoids can significantly influence amitriptyline metabolism. [10] One study showed that four different patients who were the same age as the patient in this case experienced adverse effects such as tachycardia, cognitive changes, and delirium with concurrent marijuana use while taking TCAs [11]. Regarding amitriptyline specifically, 30 mg of cannabidiol (CBD), which is a component of cannabinoids, has been shown to significantly increase amitriptyline plasma concentrations. [10] CBD has been shown to strongly inhibit CYP2C19, CYP2D6, and CYP3A, while THC inhibits CYP3A, all of which are enzymes used to also break down amitriptyline [12].

In our case, the patient on day seven had an amitriptyline level of 301 ng/mL and a nortriptyline level of 311 ng/mL, which is close to a 1:1 ratio. In the setting of cannabinoid use, this can possibly be explained because CYP2C19, which mediates the metabolism of amitriptyline to nortriptyline, and CYP2D6, which hydroxylates amitriptyline and nortriptyline, are both being inhibited. With the conversion of amitriptyline and the conversion of nortriptyline both being equally inhibited in the metabolism cascade, a 1:1 ratio would still be expected, although overall concentration levels of each will be much higher. Metabolism is influenced by many metabolic pathways and interindividual variability, so while the 1:1 ratio of amitriptyline to nortriptyline could be a possible explanation, it is important to also keep in mind other contributing factors.

Genetic and metabolic considerations

Amitriptyline is primarily metabolized by CYP2C19, CYP2D6, and at higher concentrations, CYP3A4 [3,4]. Variants in these enzymes influence whether an individual is a poor, intermediate, normal, or ultrarapid metabolizer. Poor metabolizers carry two nonfunctional alleles and may have elevated TCA concentrations and a higher risk of toxicity at standard doses [3,4]. The approximate 1:1 ratio between amitriptyline and nortriptyline levels in this case suggests that the CYP2C19 and CYP3A4-mediated pathways remained functional, making an isolated mutation here unlikely. CYP2D6 is responsible primarily for the conversion of amitriptyline and nortriptyline to 10-hydroxy-amitriptyline and 10-hydroxy-nortriptyline, respectively. A patient could have a mutation in this enzyme without impacting the amitriptyline to nortriptyline ratio since this enzyme is responsible for hydroxylating both compounds but has no impact on the conversion of amitriptyline to nortriptyline. While cannabinoid use is a possible contributing factor to prolonged toxicity, it is possible that the patient could have also had a CYP2D6 mutation.

Role of elevated body mass index

Amitriptyline is a highly lipophilic drug and widely distributed in tissues, with significant binding to plasma and tissue proteins [2]. Based on the pharmacokinetic properties of lipophilic drugs, they will have a larger volume of distribution in patients with more adipose tissue, and the clearance and half-life can be altered [13]. Studies have also shown that CYP3A4 activity decreases in patients with an elevated BMI, which is one of the enzymes responsible for amitriptyline metabolism [13]. Interestingly, there have been studies on the effect of different BMI categories on dose-corrected amitriptyline concentration levels, and it was shown that BMI has no impact on the serum concentrations of this drug [14,15]. Based on these studies, serum concentrations of amitriptyline do not change in the setting of elevated BMI, despite what would be expected based on the principles of pharmacokinetics of lipophilic drugs. The patient in this case had an elevated BMI of 36 and showed markedly prolonged toxicity levels of amitriptyline. While the use of cannabinoids is likely a contributing factor for the prolonged toxicity, it cannot be ignored that this patient had an elevated BMI. Current evidence does not consistently support a significant effect of BMI on serum levels, and its role in this case remains speculative. This case raises the importance of further investigation into whether BMI truly plays a role in altered metabolism of TCAs. While cannabinoids likely played a role, it is possible that BMI could have also been a contributing factor based on the principle that lipophilic drugs can have an altered half-life and decreased CYP3A4 activity in patients with greater adipose tissue [13].

Management considerations in TCA toxicity

Management of TCA overdose centers on rapid stabilization and prevention of complications. This case shows the importance of ECG monitoring for QRS prolongation and arrhythmias through serial ECGs every six hours. Arterial blood gas and metabolic panel to evaluate for acidosis and electrolyte disturbances, toxicology screening, and, when available, serum TCA levels are important panels to obtain. In this case, these results helped guide therapeutic management for the patient, which included continuous sodium bicarb infusion and intravenous fluids. 

Sodium bicarbonate is a cornerstone of therapy in significant TCA toxicity, particularly when QRS duration exceeds 100 ms or ventricular arrhythmias are present [9]. Alkalinization increases serum pH and sodium load, mitigating sodium channel blockade and narrowing the QRS interval. Sodium bicarbonate infusion can be tapered and discontinued once QRS duration normalizes and acidosis resolves. In this case, the patient was given continuous sodium bicarbonate until day nine when her ECG showed normal sinus rhythm. Additional measures include magnesium for QTc prolongation, benzodiazepines for seizures, and mechanical ventilation for respiratory failure, all of which were used in this patient case [8].

In this patient, serial ECGs guided ongoing therapy; sodium bicarbonate therapy was continued until the QRS duration normalized by day nine. The persistent ECG abnormalities and delayed normalization of QRS duration underscore the importance of continued monitoring in patients with increased adiposity, even when the initial clinical picture appears to be improving.

Limitations and future directions

Several limitations in this case should be considered. First, genetic testing for CYP mutations was not conducted, and therefore the possibility of a mutation contributing to decreased metabolism remains speculative and cannot be confirmed. Additionally, there was no detailed history of the patient's cannabinoid use, including frequency and duration of use, timing of use in relation to medication administration, and route of administration. This information could have strengthened conclusions regarding the impact of cannabinoid use on drug metabolism. Finally, it is important to acknowledge that the potential influence of increased BMI on the metabolism of Amitriptyline could not be directly evaluated. While some studies describe how increased BMI may impact pharmacokinetics of lipophilic drug metabolism, current evidence is not consistent in supporting a clear association. Future studies should continue to examine the relationship between increased BMI and the metabolism of lipophilic drugs to help determine whether there is a causal relationship or whether elevated BMI acts as a confounding variable. In addition, future work would benefit from more routine CYP mutation testing to better determine whether genetic variation may contribute to cases of suspected overdose or drug toxicity.

## Conclusions

This case emphasizes the importance of not only serial ECG monitoring and obtaining serum TCA levels but also considering the multifactorial nature of Amitriptyline metabolism. Certain factors such as concurrent cannabinoid use and the presence of CYP enzyme mutations could potentially influence metabolism. While some studies show elevated BMI does not play a role in slowing the metabolism of Amitriptyline, further studies should be done to confirm this, as this goes against what would be expected of the pharmacokinetics of lipophilic drugs. Overall, this case emphasizes the need for individualized, physiology-informed management of TCA toxicity in adolescents with positive cannabinoid use and other factors that can contribute to altered metabolism to reduce morbidity and optimize outcomes.
